# Evolution of photoinduced effects in phase-separated Sm_0.5_Sr_0.5_Mn_1−*y*_Cr_*y*_O_3_ thin films

**DOI:** 10.1038/srep23280

**Published:** 2016-03-22

**Authors:** Xiaojie Chai, Hui Xing, Kexin Jin

**Affiliations:** 1 Shaanxi Key Laboratory of Condensed Matter Structures and Properties, School of Science, Northwestern Polytechnical University, Xi’an 710072, China

## Abstract

Systematic study on electrical transport properties has been performed in Sm_0.5_Sr_0.5_Mn_1−*y*_Cr_*y*_O_3_ thin films illuminated by the light. An evolution of persistent and transient photoinduced effects induced by the impurity doping and temperature has been observed, which is closely related to the number of ferromagnetic clusters. The maximum persistent photoinduced effect is observed at *y* = 0.08 and the corresponding value is about 61.7% at the power density of 13.7 mW/mm^2^. The underlying mechanism can be understood by the coexistence and competition of the multiphases in phase-separated manganites induced by Cr-doping. These results would pave the way for practical applications in innovative photoelectric devices of all-oxides.

Over the past decades, doped rare-earth manganites with the general formula *R*_1−*x*_*A*_*x*_MnO_3_ (*R* is a trivalent rare-earth element and *A* is a divalent alkaline-earth ion) have attracted much interest due to their abundant physical properties and potential applications^1^,^2^,^3^,^4^. The strong coupling effects among the spin, orbital, charge and lattice often lead to an electronic inhomogeneity, which is called the electronic phase separation and usually identified with the coexistence of the ferromagnetic (FM) metal and the charge-ordered (CO) insulator^5^,^6^,^7^,^8^. The delicate balance among the diverse phases can be easily upset through “weak” external or internal perturbations and accordingly physical properties can be manipulated. In fact, the enhanced sensitivity to external perturbations, such as temperatures, magnetic fields, electric fields, optical excitation *etc.*, has been investigated in these phase-separated compounds^9^,^10^,^11^,^12^. Among these external perturbations, the photoinduced effect or optical excitation, as a contactless method, has especially drawn great attention because the energy scale of photons is much larger than that of phase transition in the solid state science^13^. As a consequence, some novel transient phases and emergent physical properties have been observed through the photoinduced interaction^14^,^15^,^16^. Early in 1990’s, Miyano *et al*. have demonstrated a photoinduced collapse of the CO state in Pr_0.7_Ca_0.3_MnO_3_ single crystals^17^. Then, Oshima *et al*. have investigated a persistent change in the resistivity upon a YAG laser without an assisting electric field in epitaxial thin films of Pr_0.5_Ca_0.5_Mn_0.96_Cr_0.04_O_3_^18^. In our previous work, the photoinduced effects in the CO La_0.5_Ca_0.5_MnO_3_ film and the La_0.67_Ca_0.33_MnO_3_ film with a typical metal-insulator phase transition, respectively, have been also revealed^19^,^20^. Heretofore, the persistent and transient photoinduced effects observed in manganites mainly originate from the different mechanisms, such as the oxygen deficiency in mostly metallic states, the melting of CO state and the photoinduced demagnetization^21^,^22^,^23^,^24^. In particular, intrinsic mechanisms need to be uncovered further. Apart from that, a partial replacement of Mn atoms by Cr ions in charge-orbital ordered manganites would result in the emergence of the FM phase and thus the two phases coexist. This could cause three fundamental effects: i) the disorder effect due to the lattice defects, which always frustrates the long range CO phase; ii) the FM double exchange interaction between Mn^3+^ -Cr^3+^ and Mn^3+^ -Mn^4+^, which induces the ferromagnetic clusters; iii) the substitution of Cr^3+^ for Mn^3+^, which reduces the local *e*_*g*_ charge density and stabilizes the antiferromagnetic (AFM) superexchange interaction^25^. Following this idea, Sm_0.5_Sr_0.5_Mn_1−*y*_Cr_*y*_O_3_ (SSMCO) are selected as the prototype phase-separated manganites in order to inquiry the CO and FM phase coexistence since the competition among the FM, CO, and AFM states becomes dominant at half doping (*x* = 0.5). Additionally, multicritical peculiarities have been observed in the electronic phase diagram, making SSMCO as an interesting candidate to reveal the phase competition and its related phenomena^26^,^27^. Therefore, we prepare SSMCO films and uncover their evolution of photoinduced effects at low temperatures. Our results demonstrate that the photoinduced effects can be tuned significantly through the partial replacement of Mn atoms by Cr ions and the temperatures.

## Results

**Figure 1:**
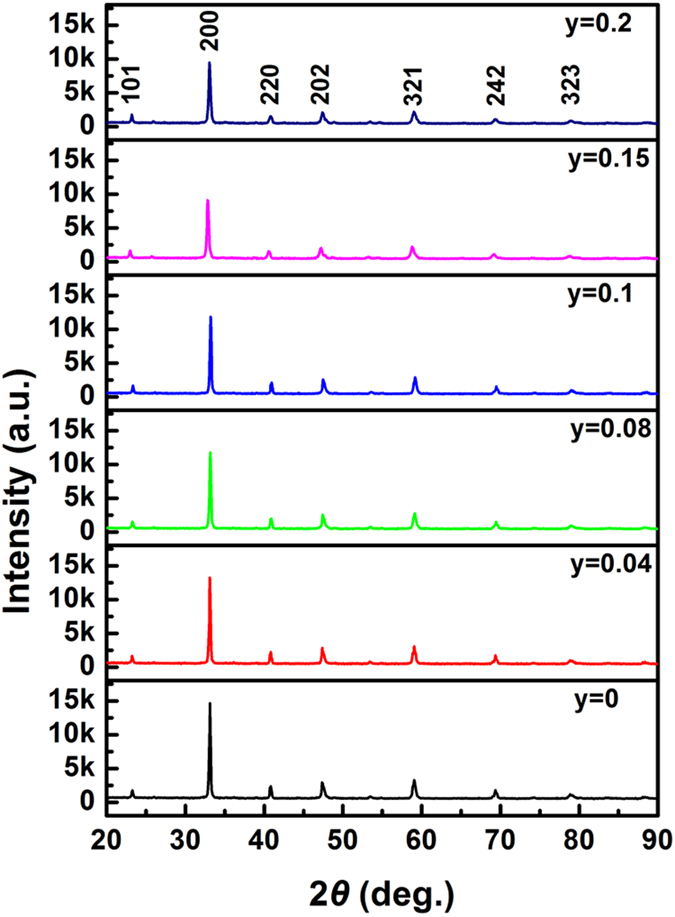
XRD patterns of the Sm_0.5_Sr_0.5_Mn_1−*y*_Cr_*y*_O_3_ (*y* = 0, 0.04, 0.08, 0.1, 0.15, and 0.2) bulk targets.

**Figure 2:**
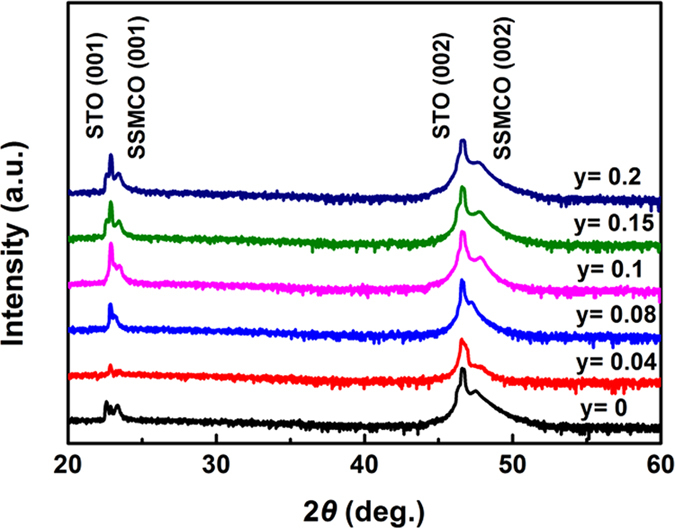
XRD patterns of the Sm_0.5_Sr_0.5_Mn_1−*y*_Cr_*y*_O_3_ (*y* = 0, 0.04, 0.08, 0.1, 0.15, and 0.2) thin films.

**Figure 3:**
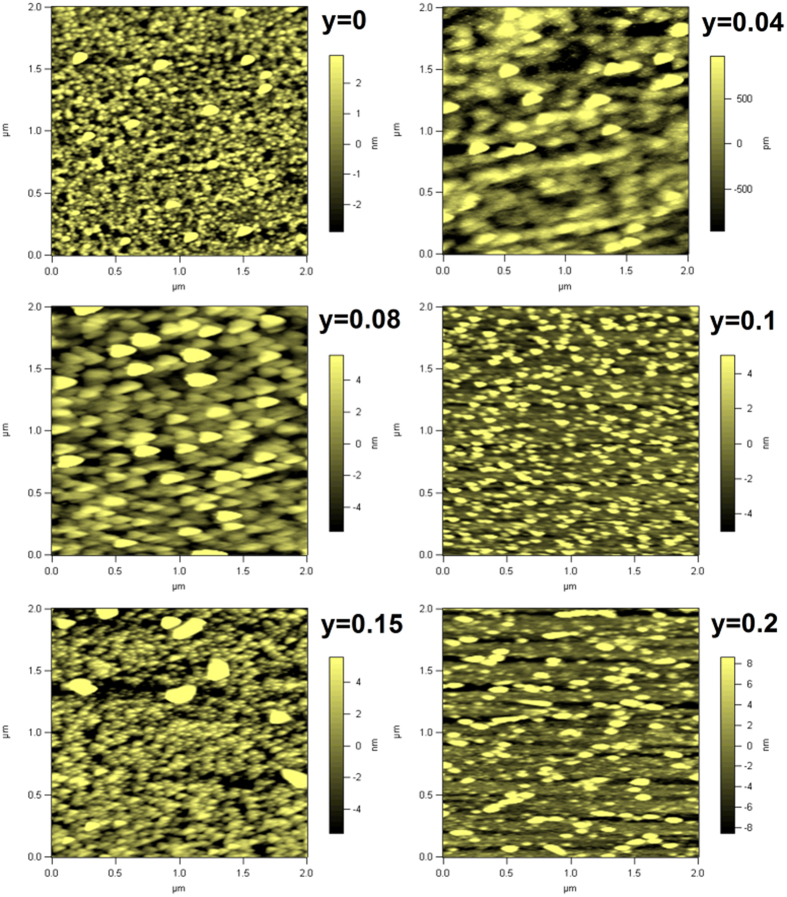
Atomic force microscopy images of the Sm_0.5_Sr_0.5_Mn_1−*y*_Cr_*y*_O_3_ films over 2 × 2 μm^2^ scan area.

As shown in Fig. 1, we present the measured X-ray diffraction spectra (XRD) at room temperature for bulk targets. Pure orthorhombic crystalline structure with the *Pnma* space group is identified and the diffraction peak positions are identical for all the samples, indicating that no impurity phases are detected with increasing the Cr doping content and the Mn ion is substituted by the Cr cation in the targets. The XRD patterns of films are shown in Fig. 2 and the intensity is given in the log scale. Besides (001) and (002) diffraction peaks of STO substrates, only (001) and (002) diffraction peaks of films appear. Thus, the films have the preferred orientation with an orthorhombic structure. Figure 3 displays the surface topographies of the films. We can see that the films with the uniform growth have the spherical, uniform, and small islands. The values of root-mean-square roughness are 1.89 nm, 0.68 nm, 3.72 nm, 3.13 nm, 4.53 nm, and 6.29 nm for the films at *y* = 0, 0.04, 0.08, 0.1, 0.15, and 0.2, respectively.

**Figure 4:**
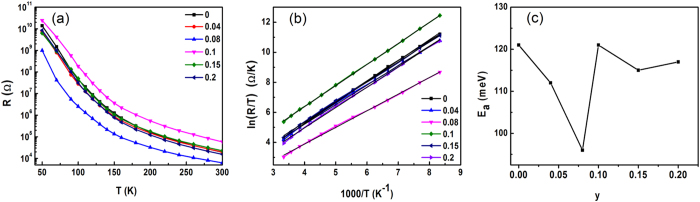
(**a**) Temperature dependence of the resistances in the Sm_0.5_Sr_0.5_Mn_1−*y*_Cr_*y*_O_3_ thin films. (**b**) The relation between ln(*R/T*) and 1000/*T* for Sm_0.5_Sr_0.5_Mn_1−y_Cr_y_O_3_ films. The straight lines represent the best fits to the Emin–Holstein model of *SPH*. (**c**) Activation energy dependence of doping concentration for all films.

Figure 4(a) shows the temperature dependence of the resistances of SSMCO films in darkness. It can be seen that all the films exhibit the insulating character over the measured temperature range. In addition, the different values of resistances are possibly caused by the residual strain. Generally, the Sm_0.5_Sr_0.5_MnO_3_ without the Cr dopant is a phase-separated system with the coexistence of the CO and FM phases^26^. Moreover, it is well-known that the Cr-doping at the Mn site leads to the melting of charge ordering as well as induces an increase of FM clusters, which usually is accompanied with an insulator-metal transition^28^,^29^,^30^. Nevertheless, this transition is dependent on the percolative characteristics, which are related with the fraction of FM clusters. Here, the FM regions are not enough to form the percolative process with the increasing Cr dopant and accordingly the transport behaviors of all the SSMCO films still show the insulating conduction. In order to gain better insights into the nature of interaction between the hopping conduction and the strength of electron-phonon interaction, we make a fit to the resistance data using the Emin-Holstein adiabatic small polaron hopping model^31^. The resistance in the adiabatic limit is given by the formula:


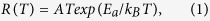


where *E*_*a*_ is the activation energy for the polaron hopping, *A* is the per-exponential and *k*_*B*_ is the Boltzmann constant. Figure 4(b) shows the inverse temperature dependence of ln(*R/T*), indicating a clear linear dependence at 120 K < *T* < 300 K. The activation energies of all the samples are estimated from the plots of ln(*R/T*) versus 1*/T*. The activation energy as a function of the doping content is presented in Fig. 4(c). The film at *y* = 0.08 has the minimum activation energy (*E*_*a*_) of about 96 meV obtained from the fitting. It is common that the partial replacement of the Mn-O-Mn bonds by the Mn-O-Cr bonds can strengthen the double exchange interaction at low doping content (*y* < 0.1), thus resulting in the carrier delocalization^30^,^32^. When the doping content is increased further, the Mn^4+^ ions are replaced by Cr^3+^ ions and the hopping probability of electrons from Mn^3+^ to Mn^4+^ becomes smaller^33^, resulting in an increase in the activation energy. Thus, the activation energy has the minimum value at *y* = 0.08.

**Figure 5:**
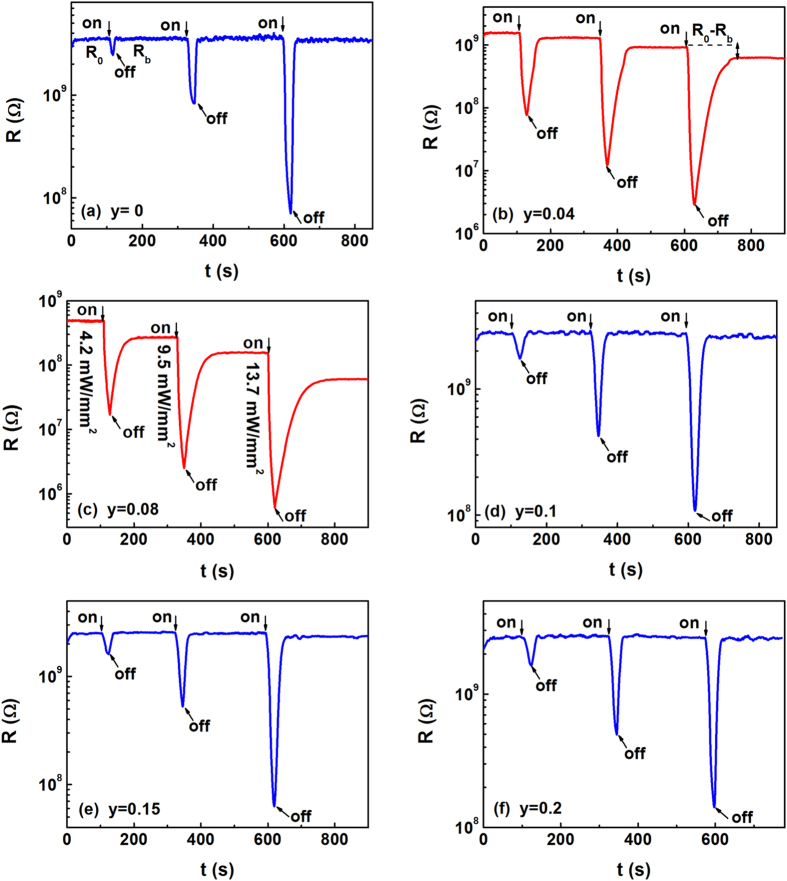
(**a–f**) Show the time dependence of the resistances of films under the light illuminations at 50 K, the marked “on an off” indicates the switching on and off of the light illumination. All the samples are illuminated by the light with three different powers, the application of illuminations of 4.2 mW/mm^2^, 9.5 mW/mm^2^, and 13.7 mW/mm^2^ with intermediate switching off.

**Figure 6:**
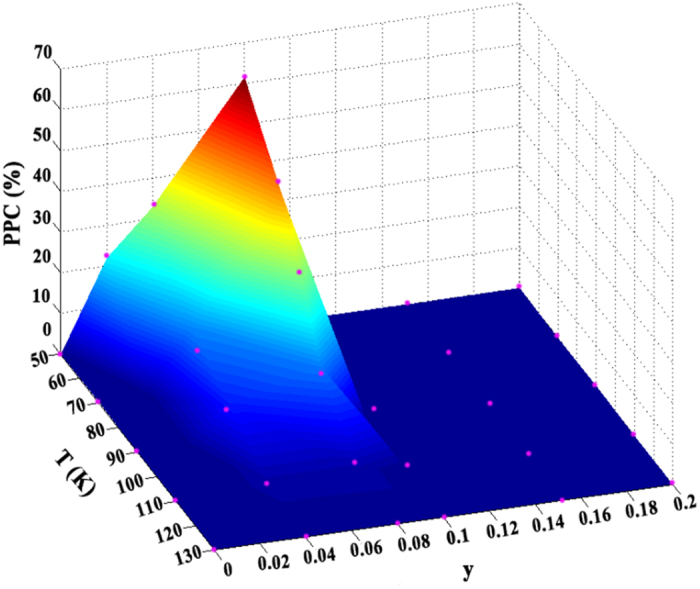
The PPC effects for the films under the light illumination at the power intensity of 13.7 mW/mm^2^ as a function of the doping contents and temperatures.

Figure 5(a–f) show the time dependence of the resistances of films under the light illuminations at 50 K, the marked “on an off” indicates the switching on and off of the light illumination. All the samples are illuminated by the light with three different powers. The power of the first light illumination is 4.2 mW/mm^2^ and the illumination time is about 20 s. After the resistance recovers to a stable value, a light with the power of 9.5 mW/mm^2^ is applied again for 20 s. And this process is repeated at the light illumination of 13.7 mW/mm^2^. Several characteristics are observed as follows. Firstly, the resistance of the film at *y* = 0 goes back to the initial value after the illumination. Nevertheless, as shown in Fig. 5(b,c), the resistance drops sharply upon the illumination of light and does not go back to the initial value when the light is turned off. This phenomenon characterizes a persistent photoinduced effects. The persistent photoinduced ratio is defined as PPC = (*R*_*0*_ − *R*_*b*_)^*^100%/*R*_*0*_, where *R*_*0*_and *R*_*b*_ are the initial resistances without the light illumination and the balanced resistance after the light illumination, respectively. If the temperature is kept at 50 K, the effect can stay for a few hours. Moreover, the PPC effects would be quenched on the thermal cycling. Namely, if the films are heated to room temperature and then cooled to 50 K in darkness, the resistances are restored to the initial values before the illumination and the PPC effects would be created again. For the films with *y* = 0.1, 0.15, and 0.2, the PPC effects disappear and meanwhile the transient photoinduced effects emerge. The maximum PPC effects appear at *y* = 0.08 and the values are about 41.4%, 44.8%, and 61.7% at the power densities of 4.2 mW/mm^2^, 9.5 mW/mm^2^, and 13.7 mW/mm^2^, respectively. This is consistent with the appearance of the minimum activation energy at *y* = 0.08 as mentioned above. Secondly, the resistances are decreased quickly with increasing the power densities and the multilevel stable values can be obtained when switching off the light. Furthermore, the PPC effect is enhanced as the power density is increased. To further understand the PPC effects, similar measurements have been performed at different temperatures for the series films. Before each measurement, the films are heated to room temperature to quench any residual persistent photoinduced effects. Subsequently, the films are cooled from 300 K to the setting temperature in darkness and then the time dependences of the resistances of films irradiated by the light are obtained at this temperature. Thus, the PPC values as a function of the temperature and the Cr doping content are displayed in Fig. 6. It is observed that the PPC effects appear for the films at the moderate doping content (*y* = 0.04, and 0.08) and lower temperatures. The magnitude of PPC effects is negligible at *T* > 130 K and at high doping region (*y* > 1.0). Therefore, the PPC effects can be tuned by the Cr doping content and temperatures.

## Discussion

**Figure 7:**
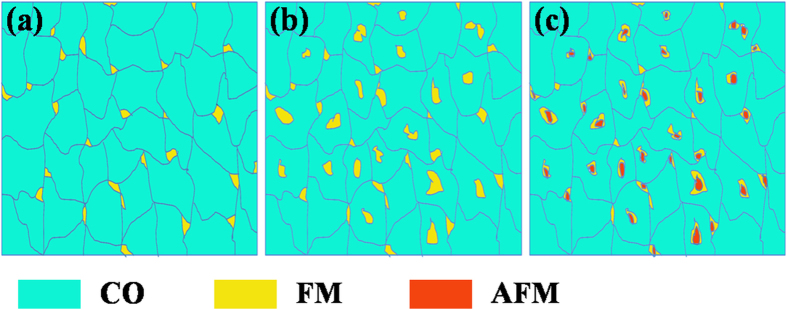
The schematic illustration of the nanoscale phase separation between FM, CO and AFM phases at different doping contents. (**a**) the film at *y* = 0, (**b**) the films with the low Cr doping content, (**c**) the films with the high Cr doping content.

It is known that different photoinduced behaviors originate from the different mechanisms affecting the electric properties of SSMCO films, which are closely related to the Cr doping effect. The light illumination would pump *e*_*g*_ electrons to transfer from Mn^3+^ ions to adjacent Mn^4+^ ions through an O_2*p*_ orbital in the FM clusters, enhancing the double exchange interaction. Furthermore, this would result in the growth of FM clusters and increase the conduction paths. When the light is switched off, the resistance recovers partly and a multilevel metastable state can be obtained with stable resistance values. In general, the FM state is comparably stable to the CO state due to the double exchange interaction with the local FM order^34^. The metastable state is stabilized by the competition between the FM and CO states. Consequently, the observed PPC effects are related to the number of FM clusters. Figure 7 displays the schematic illustration of the nanoscale phase separation in manganites at different doping contents. The pristine compound Sm_0.5_Sr_0.5_MnO_3_ is considered as a typical electronic phase separation with a multicritical point, in which the long-range charge or orbital ordering state competes with the FM state at 50 K^26^. For the films at low doping content (*y* = 0.04 and 0.08), the Cr^3+^ substitution can be viewed as a quenched random field in the charge and orbital sectors, disconnecting the correlation of charge and orbital ordering^25^. Meanwhile, the Cr^3+^ ions give rise to the FM interaction via the double exchange coupling since the Cr^3+^ ions have same electronic structure (*3d*^3^, *t*^*3*^_*2g*_, *e*_*g*_^*0*^) as Mn^4+^ ions and can be connected to Mn^3+^ ions via the oxygen in a similar way as Mn^4+^ ions. The phase competitions between the CO and FM would state would produce metastable states and the balance between these phases can be efficiently affected by the photoexcitation, thus inducing the transformation between metastable states. Accordingly, the persistent photoinduced effects occur. For the films at *y* = 0.1, 0.15 and 0.2, there is an absolute reduction in Mn^3+^ ions with the increase of Cr-doping content, which results in the decrease of FM clusters. Furthermore, the new AFM domains would come into being due to the superexchange mechanisms between the Cr^3+^/Cr^3+^ ions or Cr^3+^/Mn^4+^ ions^35^. So the number of FM clusters is decreased and as a result the PPC effects disappear. Similar reasons are exerted on the temperature dependence of the PPC effects. The FM region is shrunk with increasing the temperature, resulting in the weaker PPC effects. Ultimately, the FM clusters disappear and the films favor the *A*-type AFM state at *T* > 130 K^26^. Thus, the PPC effects are negligible.

In summary, we have systematically investigated the effect of the light illumination and the doping Cr ions on the electrical transport properties in Sm_0.5_Sr_0.5_MnO_3_ film grown on STO substrates. Above all, the film shows a transient photoinduced effects in the pristine compound. For the low doping content, the introduction of Cr^3+^ induces the creation of persistent photoinduced effects. For the films with *y* > 0.1, the FM clusters are decreased slowly with the increase of Cr-doping content and the PPC effects disappear. The evolution of the photoinduced effects is closely related with the number of FM clusters. These results can be explained by the phase competition and the instability of the multiphases in manganites. From the view of potential applications, it is very meaningful for practical photoelectronic and memory devices of all oxides.

## Methods Section

Bulk targets of Sm_0.5_Sr_0.5_Mn_1−*y*_Cr_*y*_O_3_ (*y* = 0, 0.04, 0.08, 0.1, 0.15, and 0.2) were synthesized using the standard solid-state reaction method. The SSMCO films with the thickness of about 100 nm used in this experiment have been deposited on SrTiO_3_ (STO) (001) substrates by using a pulsed laser deposition method. The temperature of substrates was maintained at 800 °C for 30 min before the deposition to obtain contamination free and flat surfaces and during the deposition in an oxygen atmosphere of 10 Pa. KrF excimer laser pulses with energy of about 130 mJ and a repetition rate of 2 Hz were focused onto the targets with a nominal composition of SSMCO. The structures and surface morphology of these films were analyzed by XRD using Ni filtered Cu kα radiation operated (RigakuD/max-2400, the wavelength is 0.15432 nm) and an Asylum Research MFP-3DTM atomic force microscope, respecticely. The electrical transport properties were measured using the multimeter (Keithley 6487) in the temperature range of 50–300 K in a closed-cycle helium cryostat with optical windows. A laser (wavelength λ = 532 nm) with variable power densities was used to illuminate the sample through the optical window of the cryostat for the photoinduced measurements.

## Additional Information

**How to cite this article**: Chai, X. *et al*. Evolution of photoinduced effects in phase-separated Sm_0.5_Sr_0.5_Mn_1−*y*_Cr_*y*_O_3_ thin films. *Sci. Rep.* **6**, 23280; doi: 10.1038/srep23280 (2016).
